# Elimination of HCV Infection: Recent Epidemiological Findings, Barriers, and Strategies for the Coming Years

**DOI:** 10.3390/v16111792

**Published:** 2024-11-19

**Authors:** Pietro Torre, Mariano Festa, Tommaso Sarcina, Mario Masarone, Marcello Persico

**Affiliations:** Internal Medicine and Hepatology Unit, Department of Medicine, Surgery and Dentistry, Scuola Medica Salernitana, University of Salerno, Largo Città d’Ippocrate, 84131 Salerno, Italy; ptorre@unisa.it (P.T.); mafesta@unisa.it (M.F.); tsarcina@unisa.it (T.S.); mmasarone@unisa.it (M.M.)

**Keywords:** hepatitis C, HCV epidemiology, HCV elimination, strategies for elimination

## Abstract

Hepatitis C is a disease for which in approximately 30 years we have gone from the discovery of the causative agent in 1989, to the introduction of direct-acting antiviral (DAAs) therapies starting from 2011, and to a proposal for its elimination in 2016, with some countries being on track for this goal. Elimination efforts, in the absence of a vaccine, rely on prevention measures and antiviral therapies. However, treatment rates have declined in recent years and are not considered adequate to achieve this goal at a global level. This poses a great epidemiological challenge, as HCV in many countries still causes a significant burden and most infected people are not yet diagnosed. Consequently, efforts are needed at different levels with common purposes: to facilitate access to screening and diagnosis and to improve linkage to care pathways. In this review, we discuss the latest epidemiological findings on HCV infection, the obstacles to its elimination, and strategies that are believed to be useful to overcome these obstacles but are applied unevenly across the world.

## 1. Introduction: An Infection Destined to End

Hepatitis C virus is responsible for a blood-borne infection that damages the liver, and the resulting chronic inflammation can lead to the development of fibrosis, cirrhosis, and hepatocellular carcinoma [1,2]. It is a major health problem, considering that it is currently estimated that around 50 million people are infected, with 1 million new infections per year [3]. The reduction in prevalence compared to previous years is attributable to the revision of previously made estimates, but also to the impact of HCV therapies [1]. Direct-acting antivirals (DAAs) have represented an important revolution in HCV management, since a short (8–12 weeks) and well-tolerated therapy cycle guarantees the cure of the infection in almost all cases. This progress, to be associated with preventive measures, and even if a vaccine is not available, has made the scientific world think that the HCV can be eliminated [2,3,4]. In 2016, the WHO released the first Global Health Sector Strategy (GHSS), referring to the 2016–2021 period, which presented the goal of eliminating viral hepatitis as a public health threat by 2030. This document concerns all five hepatitis viruses (hepatitis viruses A, B, C, D, and E) but mainly refers to HCV and HBV, due to the impact they have on public health. The strategy is structured in five main components and includes a series of fundamental actions to be undertaken by countries and by the WHO itself. Subsequently, in 2021, an interim guidance for the validation of elimination was published, which redefined the targets to reach [5,6]. In 2022, a new GHSS covering the period 2022–2030 was published. In this case, a common vision for the end of HIV, viral hepatitis, and sexually transmitted infections was proposed, placing emphasis on the needs of individuals and communities. As these diseases share many aspects, including transmission routes and key elements for their control, this document provides both shared and disease-specific actions to pursue [7,8]. HCV elimination, however, is hampered by several barriers, which ultimately result in suboptimal diagnosis and treatment rates [9,10]. Moreover, it requires constant monitoring of its progress, which is not an easy task. For this purpose, together with the epidemiological monitoring of hepatitis B, the Polaris Observatory (a Center for Disease Analysis initiative) was created in 2015, which offers a simple online interface to consult which countries are “on track” for elimination, that is, when absolute or relative impact and programmatic targets are met [6,9,11,12]. In this review, we discuss recent epidemiological findings around the world, barriers to elimination, and strategies that could overcome them and enhance HCV screening, diagnosis, and treatment.

## 2. Where HCV Infection Persists: General Population vs. Key Populations

Although it is known that there is a large disparity in the burden of HCV between countries, but also within the same country, its real spread is unknown, because prevalence studies often provide incomplete and inaccurate pictures, and this infection is associated with a paucity of manifestations, until the liver disease is in an advanced stage. Moreover, the spread of hepatitis C varies also in the same setting depending on temporal and socio-economic factors. These aspects make it difficult to target interventions and allocate resources. Universal screening might seem like a solution to this problem and proved to be the most effective strategy in specific conditions. In the United States, the CDC currently recommends HCV screening for all adults aged ≥18 years at least once and for all pregnant women, except in settings with prevalence <0.1%, and regardless of age and prevalence for people at risk of infection. However, costs and structural problems make its large-scale implementation unfeasible [1,12,13,14,15,16,17]. The concept of “micro-elimination” was an attempt to reduce the impact of the HCV epidemiological challenge and consists of concentrating elimination efforts, including case finding, on specific contexts (e.g., addiction centers, prisoners, hospitals, residents of specific geographic areas, specific age groups, people with HIV, and others) [18]. Today, while HCV testing in “key populations” (a term that has been used to include several risk categories, but used in this case in particular for people who inject drugs (PWID) and prisoners) is widely recommended, there is still heterogeneity, research, and discussion about the best screening target in the general population, even in the same country [17,19,20,21].

### 2.1. General Population

HCV infection in the general population has spread for different reasons in different parts of the world. Before its characterization (1989) and subsequent systematic testing, transmission was linked to transfusions of blood and its derivatives, dialysis, or other medical interventions, such as dental procedures, using contaminated equipment. Among recurring examples of healthcare related transmission there is also the mass parenteral therapy for Schistosomiasis, which took place decades ago in Egypt. In recent years, thanks to HCV testing, these modes of infection have been reduced. For this reason, today, HCV infection in the general population is mainly found in elderly subjects [22,23,24,25,26,27,28,29,30]. However, while medical transmission has decreased over time in high-income countries (HICs) it continues to be an important problem in low- and middle-income countries (LMICs) [23,31]. It was estimated that unsafe medical injections globally contribute to 13.8% of new HCV infections, with significant variation depending on the area and with the highest burden reported in the Southeast Asia Region. Pakistan, where medical injections are widely used, is estimated to contribute >40% of new infections related to this modality [32,33,34]. Furthermore, although nucleic acid testing (NAT) for blood donation has increased over the years, the economic aspect is an important barrier to its implementation in LMICs [23,35,36]. In addition to healthcare-associated contagion, HCV can spread in the general population in other ways. Regarding sexual activity, HCV is transmitted with a low efficiency, lower than that of other viruses such as HBV and HIV. In this context, HCV spreads across mucosal surfaces through the exchange of blood, and while in stable heterosexual couples the risk is very low, it rises in people having sexual intercourse with multiple partners, unprotected anal sex (e.g., men who have sex with men, MSM), or other sexually transmitted diseases (STDs) [1,37,38]. Vertical transmission, that is, transmission from an HCV-RNA-positive mother to her child, occurs in a percentage of approximately 5–6%, and higher if HIV infection is also present [39]. Tattoos and piercings (which have increased over the years) can also be risky practices, as well as sharing contaminated toothbrushes and razors [16,23,40]. The general population is the setting where case finding has represented the greatest challenge, and the screening strategies that have been adopted vary across countries and have changed over time. In addition to the universal screening mentioned above, recommendations in most cases focus on people with risk factors for HCV infection. Another strategy is birth cohort screening, which consists of testing people based on year of birth, regardless of knowledge of specific risky exposure, given their overall greater likelihood of being infected compared to other cohorts. Although with slightly different age limits, age-cohort-based screening most frequently targets older age groups (e.g., 1945–1965) [16,41,42,43,44]. However, there are also exceptions, for example, in Italy, based on cost-effectiveness studies, free screening has been adopted for a younger cohort (1969–1989), with the intention of later extending it to older people as well. This approach could also be useful for countries that cannot afford universal screening, but its implementation should consider the local HCV epidemiology [1,17,45].

### 2.2. Key Populations

An estimated 15.6 million people use injection drugs globally, and the prevalence of HCV infection in this category is very high, largely variable depending on the subset considered. Injection drug use (IDU) is the main risk factor for new HCV infections in Western regions, such as North America and Europe [28,34,35,46,47,48]. In the United States, the increase in acute HCV infections has been related with the rising opioid epidemic [49]. Considering these aspects and the global epidemiology of HCV, drug use has been conceived as a leading risk factor for hepatitis C in low-prevalence countries (HICs) while playing a minor role in LMICs, some of which host a high prevalence of infection [31,50,51]. It was estimated that globally, in the period 2018–2030, 43% of new HCV infections could be prevented by removing the elevated risk of transmission related to drug use, ranging from 14% in sub-Saharan Africa (which nevertheless continues to have a high prevalence of infection, with a complex and difficult-to-investigate scenario) to 96% in Eastern Europe [23,50,52]. Recent findings, however, document that IDU is increasing also in LMICs, and a large burden of IDU-related HCV infections is found in some Asian and South American countries [1,34,53,54,55,56]. It was also reported that, unlike in LMICs, HCV infection is decreasing in some HICs, thanks to risk reduction policies and antiviral therapies, although it is difficult to draw reliable conclusions based on the available data [57]. At the time of presentation for therapy, PWID are younger compared to the general population, and they are also predominantly male and with a lower burden of significant fibrosis or cirrhosis [22]. In this category, contagion occurs through the sharing of syringes, needles, or other non-sterile accessories for the injection. HCV can survive in syringes for weeks, but this varies depending on the syringe (those with a high void volume are more at risk), the preference of which differs according to the area, and the environmental conditions. Several factors contribute to increasing the risk of acquiring hepatitis C in this setting, including a longer duration of drug abuse, greater frequency of injections, low level of education, poverty, injections in outdoor spaces, and unstable housing. Infected PWID can also spread the infection through sexual transmission [48,58,59,60,61,62,63,64]. A specific category with a high prevalence of HCV infection mainly related to IDU is represented by prison inmates. In this setting, HCV antibody positivity estimates are variable and depend also on the geographical area [65,66]. Already infected individuals can import the infection into prison, and for uninfected, incarcerated people, there is the risk of contagion during their stay in the penitentiary. Here, in a context where it is difficult to apply risk reduction behaviors and access sterile equipment, they could also have their first experience with drugs. Once released, they can spread HCV into the community, fueling its circulation [65,67,68,69].

## 3. Barriers and Strategies for HCV Elimination

Despite the availability of effective antiviral regimens, it is estimated that HCV elimination will not be achievable within the targeted timeframe in most countries mainly due to suboptimal diagnosis and treatment rates. Another problem is when HCV infection is discovered or treated when advanced fibrosis or cirrhosis has already set in [9,12,70,71]. In this perspective, the path to elimination, although characterized by a reduction in prevalence over the years, seems to become increasingly difficult as time progresses, and changes and adaptations appear necessary. These should address what is behind the current insufficient capacity to manage hepatitis C, such as the lack of infrastructure and healthcare personnel, the complexity of treatment rules, the costs and time required for the assessment of the disease, and costs of drugs, which have a different impact depending on the area of the world. Therefore, standard measures to be applied to contain HCV infection, political commitment, and cost-saving strategies for diagnostic tests and DAAs must be combined with new modes of action, such as decentralization, the integration of services, and the simplification of diagnosis and treatment. These changes may be especially useful for groups who have a difficult access to HCV care, such as people living in remote areas or in LMICs, migrants, PWID, or prisoners [8,9,22,30,72,73,74,75,76,77,78,79].

Decentralization consists of moving screening and treatment from tertiary or specialist sites to peripheral facilities or primary care, addiction services or prisons, with the aim of providing HCV management closer to patients. This is adopted by many countries that are doing well in the fight against HCV and is in line with the “people-centered” model of care supported by the WHO. The rationale of this strategy is that providing HCV treatment in tertiary centers may represent a significant barrier, either physical or non-physical, with the latter due to the socio-cultural gap that may exist between disadvantaged groups and the staff of specialized centers [8,80,81,82,83,84]. A recent systematic review and meta-analysis highlighted the benefits of a decentralized model of care with respect to linkage to care and treatment uptake. Moreover, sustained virologic response (SVR) rates were high regardless of the level of decentralization and therapy provided by non-specialists (task-shifting), including primary care physicians (PCPs) was associated with similar SVR rates compared with therapy provided by specialists [85]. The latter is another fundamental aspect to consider to expand the access to therapy, but in a 2024 study investigating DAAs restrictions around the world, the most common restriction turned out to be who can prescribe DAAs, which means, who can treat hepatitis C, with 61% of countries having a specialist-only prescribing policy [56]. This problem must be overcome, also because the currently available drugs are extraordinarily effective and almost free of side effects, making the specialist expertise in most cases not necessary [84].

PCPs should be a prominent part of a decentralized network of care, as they represent the first approach to the healthcare system in most cases, and ideally, they are involved in screening and prevention activities. However, because they are often overworked and have limited resources, they may feel discouraged in dealing with hepatitis C. For this reason, interventions aimed at informing and training on HCV infection diagnostic assessment and treatment (task-sharing), as well as, in general, at strengthening the network between PCPs and liver specialists, must be implemented [45,56,84,86,87,88,89,90]. Pharmacies and pharmacists represent further potential, as they are spread out all over the territory, work in close contact with people (both general population and drug users), and have already demonstrated their potential in disease testing. However, the same barriers (work overload and poor knowledge of the disease) and possible solutions as those for PCPs could partly apply [91,92,93,94,95,96]. Mobile units (offering point-of-care, POC, HCV testing, pre-therapy liver disease severity assessment and treatment), or home self-testing could be useful for people who cannot reach conventional sites for geographical or other issues. In any case, a valid model of person-centered care requires efficient interaction between the different actors, which can be facilitated through an innovative use of technology [84,86,90,97].

The concept of integration is related to decentralization, which means co-localizing and integrating hepatitis C testing or therapy with other services for which people go to a specific site. This integration, which helps reduce the fragmentation of care pathways, could be applied to the context of addiction services, prison, general practice, and HIV clinics [84,86,87,90,98,99,100,101]. Opportunistic HCV screening, such as that recently carried out during the SARS-CoV-2 pandemic, and exploiting its related services, or performed in hospitals, for example, in emergency departments (EDs), would also aim to increase testing opportunities [19,102,103,104,105,106]. This could be advantageous because it leverages the infrastructure of other services, and no active recruitment policies for people to be tested or extra staff may be needed. However, it may be limited by the fact that not all people at risk for HCV infection, for various reasons, undertake activities associated with screening [105,107].

The simplification of diagnosis and treatment algorithms are also useful strategies and are often part of decentralized models of care. The simplification of diagnosis may be based on the use of POC tests, which are also called rapid tests. They include salivary tests and tests using capillary blood by fingerstick to look for HCV antibodies, which allow results to be obtained in about 20 min. Today, POC HCV-RNA tests are also available, which, with samples obtained by fingerstick or venipuncture, and giving a response in about 1 h, could allow the diagnostic cycle to be concluded at the time of the visit (“test and treat” model). Moreover, it can also be used to assess the response to treatment. Another way to simplify diagnosis is reflex testing, that is, looking for HCV-RNA in people who tested antibody-positive using the same blood sample sent for the antibody test or two samples (the first aimed at the detection of antibodies, followed, if positive, by another sampling to search for HCV-RNA, through POC or lab-based testing), but in a single visit. These approaches reduce the time to treatment initiation, which could be weeks according to conventional protocols, and could also be useful in cases of patients with difficult access to the veins. Dried blood spot (DBS) is another diagnostic possibility and consists of releasing a few drops of blood onto absorbent paper that is then sent for analysis for the identification of HCV antibodies or RNA [1,30,73,75,84,86,108,109,110,111]. The latest recommendations for the management of HCV infection by the EASL (European Association for the Study of the Liver) and AASLD (American Association for the Study of Liver Diseases) are also in this direction. These guidelines, moreover, suggest that HCV replication is the main information to obtain in a simplified model of care, bypassing genotype testing and using simpler methods than liver stiffness measurements for the identification of F3 or F4 patients, such as FIB-4 and APRI [75,112].

These new modes of action may be particularly useful for difficult populations, including PWID, which represent a complex group to reach, treat, and keep in contact with for health facilities, due to their socioeconomic and other problems such as the risk of reinfection, psychiatric comorbidities, health insurance issues, and the stigma they may experience when they encounter the health system. For incarcerated subjects, there is also the issue of possible release and loss of contact with the system of care. Due to these problems, PWID were considered suboptimal candidates for antiviral therapy. However, the overall cure rate for PWID is very high, and they are now considered a category on which to concentrate great efforts. Treatment uptake, however, is still low for them and must be increased to achieve HCV elimination [10,56,73,75,84,113,114,115,116,117,118]. This category requires systematic screening followed by treatment, which should take place in a decentralized and simple way, also using telemedicine. In parallel to this, interventions aimed at risk reduction (counselling, opioid substitution therapy (OST), and needle and syringe programs (NSPs)) are strictly necessary, and the co-localization of these services with HCV testing and treatment could further improve the results [3,34,46,65,117,119,120,121,122,123,124,125,126,127,128].

**Figure 1:**
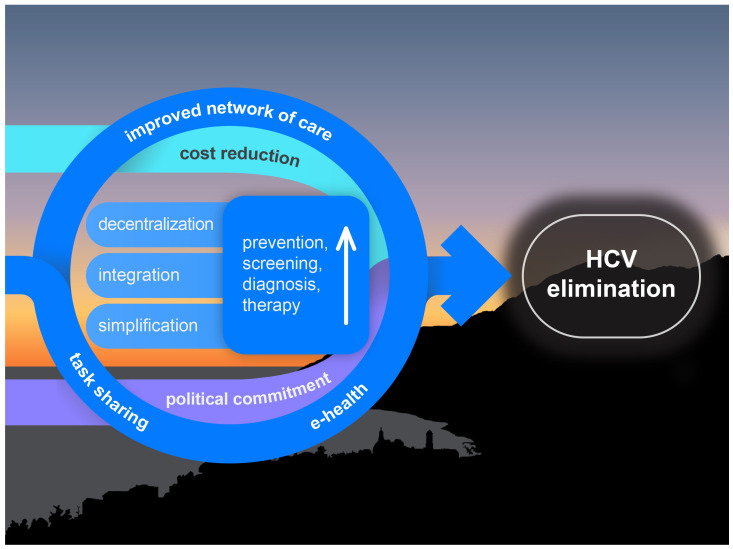
The “sunset boulevard” of hepatitis C: the HCV elimination process requires a paradigm shift to make hepatitis C care widely accessible.

Figure 1 depicts the elements discussed in this section, which together could enhance hepatitis C prevention, screening, diagnosis, and therapy.

## 4. Telemedicine and Informatics to Support HCV Elimination

The recent SARS-CoV-2 pandemic served as a catalyst for the use of telemedicine, which was also useful in this case to avoid contagion. The hepatological societies released recommendations in that period that defined its specific cases of application [129,130]. It has demonstrated its usefulness in the management of patients with hepatitis C in the general population and in key populations during those years, but it also did so before [131,132,133,134,135,136,137,138,139]. The rationale for its use is mainly linked to situations in which, for patient- or physician-related reasons, it is not possible to carry out a traditional visit (including long distances and/or conditions that limit travel) [140]. In addition to classic telemedicine, i.e., the patient–physician interaction, other models were also explored, such as the ECHO (Extension for Community Healthcare Outcomes). This was developed by the University of New Mexico Health Sciences Center (UNMHSC) with the aim of improving access to specialized care in underserved areas or prisons of New Mexico, and the first disease to be managed was hepatitis C. This model consists of virtual meetings, including real cases of patients to be managed, between experts of a specific condition and community providers. The latter, in this way, are trained to treat a specific condition, with the added benefit of potentially overcoming the cultural barrier that might exist between the staff of tertiary or university centers and the population of a given location. This approach is considered to save time and have the ability to increase treatment capacity [76,82]. In a study comparing the efficacy of ECHO-based hepatitis C treatment with treatment at the University of New Mexico (UNM), similar SVR rates were found between the two cohorts [141]. This model was then implemented in the Department of Veterans Affairs (VA), was applied in other countries, and even in the DAAs era [73,82,142,143]. In addition to telemedicine, technology could be useful in the management of HCV in other ways. For example, through prompting the identification of patients at risk of infection, or who have not previously been tested, and to be referred for screening, or by facilitating the referral of positive patients to specialists, by flags, notes, or other types of alerts or reminder integrated into informatic systems [144,145,146,147,148,149,150,151].

## 5. An Overview of Countries That Are on Track for HCV Elimination

As mentioned, we are currently far from the diagnosis and treatment targets set by the WHO. Underdiagnosis and undertreatment are not a single problem, because often, even after hepatitis C is diagnosed, treatment is not carried out. It was estimated that in HICs, about 45% of HCV infected patients were diagnosed, and only 5% were treated, while in low-income countries, about 16% of patients were diagnosed, and less than 1% were treated in 2020 [9,12]. The global decline in the number of therapies of recent years has been influenced by the SARS-CoV-2 pandemic, but it is also linked to other causes, including the Egyptian program almost concluded and the exhaustion of the pool of patients with known hepatitis C already diagnosed (and treated). The latter, given the estimated high number of people still infected, a large percentage of whom with severe liver disease, must push for the adoption of innovative methods to improve the cascade of care [12,22]. According to Polaris, 12 countries, largely different based on socio-economic factors and HCV epidemiology, are considered on track to meet the WHO targets (Australia, Austria, Denmark, Egypt, England, Finland, France, Iceland, Norway, Saudi Arabia, Spain, and the United Kingdom). This list, before the last update made at the time of the submission of this paper (October 2024), included Canada, Malta, and Rwanda, which will also be discussed. Some countries, such as Italy, while previously on track, are currently not, probably due to estimates distorted by the high number of therapies prescribed following the adoption of universal access (that is, the possibility of DAAs prescription not only for patients with more severe disease), which was subsequently not maintained [1,4]. In the brief descriptions of the countries presented below, in addition to the differences, many similar aspects can be noted.

In Australia, a change in HCV infections in recent years has been observed and linked to risk reduction strategies adopted in high-risk contexts, the unrestricted access to treatment, and the expansion of prescribing capacity. In the latter aspect, Australia has been an exemplary country, as since 2016, all clinicians can prescribe therapy for hepatitis C [152,153,154]. For years, efforts have been made to inform and train physicians with no experience of treating this disease, and there are online resources with simplified indications on its management. These interventions have favored the screening and management of hepatitis C by general practitioners (GPs), who, in the initial period, are required to carry out a consultation with a specialist to prescribe DAAs [155,156,157]. These changes have been driven by several national strategies that have been released over the years. In Australia, among the categories at risk of HCV infection, on which it is recommended to employ efforts, there are also Aboriginal and Torres Strait Islander people, who are the targets of screening and treatment efforts. However, as has happened in other countries, Australia has recently faced a decline in diagnoses and treatments. This is a cause of concern and must lead to strengthening or expanding simplified models of testing and care to stay on track toward elimination [155,158,159].

In Austria, as in other European countries, fibrosis-based restrictions for DAAs were removed, although in this case, non-specialists cannot prescribe antiviral therapy. In this country, part of a low-prevalence area of Europe, most of the burden is related to intravenous drug use and to risky sexual activities. Here, initiatives aimed at simplifying the management of PWID have been implemented, including the co-localization of OST and DAAs therapy. However, neither a specific action plan nor updated data are available in this country [56,160,161,162]. Several micro-elimination initiatives were conducted in high-risk settings, and the results of a macro-elimination (ELIMINATE) project have recently been published that proved to connect patients with a previous finding of positive HCV-RNA and “lost to care”, to treatment [163].

Canada faces several challenges in managing HCV infection. Here, the different jurisdictions have heterogeneous reimbursement criteria and methods for the management of hepatitis C (with respect to fibrosis and genotype assessment, use of POC testing, and prescribers), which in some cases could limit access to treatment. It was estimated that there may be differences in achieving the elimination in different territories. Following the Pan-Canadian Framework for Action, in 2019, Canada released a Blueprint that highlighted the needs and provided information about the ongoing elimination process. It identified six categories on which to focus efforts: PWID/PWUD, indigenous, prisoners, people from areas with high HCV prevalence, gay/bisexual/MSM, and the 1945–1975 birth cohort [115,164,165,166,167,168]. From this country, several examples of strategies aimed at reaching specific populations and innovative models can be provided. In Ontario, the Hepatitis C Teams target marginalized populations such as PWID and homeless people, while in Saskatchewan, efforts have been directed at screening and treating Indigenous communities. In addition, innovative systems such as the ECHO program and others for epidemiological monitoring were also adopted. For example, the British Columbia Hepatitis Testers Cohort (BC-HTC) is a system that aims to monitor HCV epidemiology, including assessing disparities in disease burden and access to care. Another innovation is the Canadian Hepatitis C Network (CanHepC), a system that connects researchers, trainees, and other figures working in the hepatitis C field in Canada [115,169].

The case of Egypt has made headlines for its extraordinary success: in about 10 years, it went from being one of the countries with the highest prevalence of hepatitis C in the world (about 10%) to be the first in reaching, in 2023, the “gold tier” status on the road to elimination [170,171]. To achieve this goal, political, economic and people efforts have been concentrated. Egypt’s high prevalence of HCV infection has been linked to mass parenteral antischistosomal therapy (PAT), which was practiced in that area until 1980s [172]. The National Committee for Control of Viral Hepatitis (NCCVH) was created in 2006 to coordinate the efforts against this virus, and the National Network of Treatment Centers (NNTC) to connect treatment centers and the head office. A national elimination campaign was launched in 2014 and strengthened in 2018, with the 100 Million Healthy Lives campaign resulting in an event of exceptional magnitude in the history of this disease. Screening, for which trained staff were involved, was carried out in various settings, from universities to rural health units, and to increase testing capacity, mobile teams were deployed on special vehicles; the efforts were also conducted 7 days a week and 12 h a day and were supported by an intense advertising campaign. In addition to price negotiation for tests and drugs, to further reduce costs, the local manufacturing of DAAs took place. Another technological innovation was the creation of an online site for registration and booking appointments for hepatitis C care (with a very simple interface). All these efforts resulted in more than 50 million Egyptians screened and more than 4 million patients treated for HCV from 2014 to 2020 [171,173,174,175,176].

France, like other Western European countries such as Germany and Belgium, is historically considered a low-prevalence area of hepatitis C. Here, epidemiological studies have documented a progressive reduction in the incidence and prevalence of HCV infection over the years, attributed, in addition to the death of infected patients, to improved preventive measures and to increased response to treatment [162,177,178]. As an innovative strategy, we can mention a 2016 screening directed to the general population in which a home DBS testing kit for HCV, HBV, and HIV was sent to participants’ homes, showing good results [179]. Beyond the universal access to DAAs, which became effective in 2017, other important actions in facilitating the management of HCV were the hepatitis C testing simplified and implemented in different settings, DAAs dispensing by retail pharmacies, and the prescription of these drugs also by non-specialists from 2019. Given the progress made, France committed to eliminating hepatitis C by 2025 [162,178,180,181].

The Republic of Malta is in Southern Europe and is one of the smallest states in the world. At the same time, it is estimated to have one of the smallest populations affected by HCV infection (an absolute number of about 1000 people) [182]. Despite this, it has a strategy for the elimination of hepatitis C, referred to the period 2018–2025 [183].

In Rwanda, where HCV prevalence was previously estimated to be about 4%, drug use is rare, and hepatitis C has also been associated with traditional operations and scarification. This country launched a plan for HCV in 2014 and in 2018 implemented a national program aimed at HCV elimination, and like in Egypt, a massive and widespread deployment of forces occurred [184,185,186]. In this, Rwanda was an exemplary country in sub-Saharan Africa. Through decentralized, simplified, and innovative models of care (e.g., the use of rapid diagnostic tests and DHIS2 for patient tracking), cost-cutting policies, political and community support and by leveraging an already existing HIV network, the country has increased its capacity to diagnose and treat hepatitis C. By 2022, about 7 million people were screened, and 60,000 people received treatment. Models estimated that Rwanda could achieve elimination by 2030 [101,187,188], but as of the latest Polaris update, this country is no longer on track for this goal.

Saudi Arabia is part of the Eastern Mediterranean region, which is considered to have the highest prevalence of HCV infection. However, most of this region’s burden was related to infections in Egypt and Pakistan [3,189,190]. In Saudi Arabia, HCV transmission has been linked mainly to medical procedures, as transmission due to drug use was thought to be very rare. In this country, until a few years ago, the vast majority of infected patients were estimated to be undiagnosed [191]. Guided by a national program implemented by the Ministry of Health (MOH), this country applied a decentralized and simplified model of testing and care. A campaign to raise awareness of the disease was also launched (Give Your hand) [192]. Updated epidemiological data revealed a lower prevalence than previously estimated, and now Saudi Arabia is considered on track for elimination [193].

In Scandinavian (or Nordic) countries, the prevalence of hepatitis C is low in the general population, and IDU contributes the highest percentage of HCV infections, reaching estimates of 97.4% in Denmark and 95.9% in Finland in 2019. Another problem in these countries could be HCV infection in migrants coming from high-prevalence areas. Denmark, Finland, Iceland, and Norway are currently considered on-track for HCV elimination. In these countries, which can rely on adequate health systems and resources, decentralized models of care are also applied, including treatment in addiction centers or prisons, but not by GPs [162,182,194]. Among the projects in this territory, in Southern Denmark, the C-free South project aimed to diagnose 90% and treat 80% of HCV infected persons by 2025, by interventions in high-risk settings (drug addiction centers and prison) according to a “test and treat” model, recalling patients who have missed to care, HCV screening in specific settings (such as EDs and psychiatric wards), and the use of a mobile clinic to reach marginalized populations for information and testing [195]. In Iceland, the TraP HepC (Treatment as Prevention for Hepatitis C in Iceland) project was launched in 2016 and designed to upscale the testing and treatment of HCV patients (prioritizing PWID) through a multidisciplinary team. In 3 years, 95.3% of diagnosed people were linked to care, 96.5% of which were treated [196,197]. Norway also invested in projects targeting PWID, and a reduction in HCV-RNA prevalence was documented in this category over time. In this country, preventive measures to be adopted for drug users exceeded the standards set by the WHO and are among the highest in the world (over 70% of PWID are in OST, and the syringes offered exceeded 400 per person) [198,199,200,201,202].

Spain is part of Southern Europe, which includes Italy and Greece among other countries, and has been considered a high-HCV-prevalence area [162]. An action plan for controlling hepatitis C was released in 2015, and universal access to DAAs took place in 2017. Spain has treated a large number of patients (more than 165,000 in the period 2015–2023) and was estimated to have the highest treatment rate in Europe, although hard-to-reach populations such as drug users and migrants represent a major challenge. Spain, in fact, has a large population of migrants and refugees, who, depending on their characteristics, may have a high risk of infection [203,204,205,206,207,208]. In this country, innovation such as decentralization, simplification, integration, opportunistic screening, or telemedicine for the management of hepatitis C were used in high-risk settings (in this regard, JAILFREE-C study, targeting prisoners, and the Hepatitis C Free Balears study, targeting people who use drugs, can be mentioned), but also in other contexts, such as EDs [204,209,210,211,212,213,214].

In the UK, improved testing and increased access to DAAs have been linked to a reduction in the number of people with HCV of more than 47% from 2015 to 2021, with consequent reduction in mortality. The current estimated prevalence of HCV infection is around 0.2%, with the highest share of infections linked to intravenous drug use, as in other Northern European countries [126]. NHS England has invested a lot of effort in point-of-care and decentralized testing, both in the general population and in high-risk settings. Peer support has made a big contribution, and agreements with pharmaceutical companies were not only aimed at price negotiations but also at implementing elimination initiatives [150]. An important step was the establishment of 24 regional operational delivery networks (ODNs) devoted to HCV management by working in coordination with specialists, primary care, addiction services, and prisons [215,216,217,218]. GPs are an integral part of the elimination process; among the tools they can rely on is Patient Search Identification (PSI), which helps identify patients for testing, and eventually treatment, based on their risk of being infected. Other recently adopted initiatives include the screening for blood-borne viruses in several EDs, and the possibility for the population to obtain a testing kit for HCV at their own home [150,217,219]. Given the progress made, NHS England set the target of eliminating hepatitis C by 2025.

## 6. Conclusions

The hepatological community is now projected to eliminate HCV, but to achieve this result, avoiding an excess of burden due to cirrhosis, hepatocellular carcinoma, and liver-related mortality, it is necessary to increase diagnosis and treatment rates. This requires a reorganization of the hepatitis C management paths, in support of an expansion of testing and treatment capacity. It is now clear that this reorganization should correspond to a shift from an exclusively specialist model to another in which HCV diagnosis and therapy are provided across the territory according to a decentralized model of care. Political actions, national plans, and agreements with pharmaceutical companies, as well as innovative technological solutions, can shape this change together.
